# The Influence of Different Ultrasonication Parameters on Physicochemical Properties and Secoiridoid Compositions of Olive Extracts: A Mathematical Approach Using Artificial Neural Network (ANN) and Response Surface Methodology (RSM)

**DOI:** 10.3390/foods15091507

**Published:** 2026-04-26

**Authors:** Ayşe Nur Aktay, Onur Ketenoglu

**Affiliations:** Department of Food Engineering, Faculty of Agriculture, Eskisehir Osmangazi University, Eskisehir 26160, Türkiye; aysenuraktay98@gmail.com

**Keywords:** olive, ultrasound, secoiridoid, artificial neural network, response surface methodology

## Abstract

The effects of different ultrasound parameters on some physicochemical properties and secoiridoid compositions of olive extracts were investigated. For this purpose, pH, acidity, photometric color index (PCI), total phenolic content, and secoiridoid phenolic compound composition analyses were carried out in olive extracts obtained by ultrasonic extraction at different operating parameters such as temperature, ultrasonic power, and extraction time. The data obtained were modeled and optimized by using the Box–Behnken design of RSM. Then, the comparison of experimental data versus mathematical estimations was performed by using both ANN and RSM. The results revealed that the pH values of the samples ranged between 4.94 and 5.23, and the average acidity value was 0.551 (% oleic acid). PCI values varied between 20.46 and 83.70. Total phenolic content ranged between 0.13 and 0.42 mg GAE (gallic acid equivalent)/g extract. Regarding secoiridoid phenolics, the ranges for oleuropein, oleacein, and oleocanthal were 5.33–34.39 ng/μL, 0.76–6.03 ng/μL, and 3.77–14.16 ng/μL, respectively. The optimized temperature, time, and ultrasonic power were 43.13 °C, 15 min, and 100% (of the maximum ultrasonic power of 90 W), respectively. The overall desirability of the process was obtained as 95.51%. RSM and ANN were both favorable in the estimation of experimental data with slight differences.

## 1. Introduction

Olive (*Olea europaea* L.) and its extracts, in particular extra virgin olive oil (EVOO), are commonly considered as key factors in a healthy Mediterranean diet. Many health benefits have been credited with olive and olive oil consumption due to their phenolic content including a protective role on cancer [1,2,3], anti-inflammatory activity [4], antioxidant activity [5], Alzheimer’s disease and brain aging [6], and antifibrotic effect [7].

Olive plants are known to be rich in phenolic compounds at varying concentrations from 1 to 3%, including subgroups such as phenolic acids, phenolic alcohols, flavonoids and secoiridoids [8]. Among all phenolic groups, a priority has been recently given to the secoiridoids due to their bioactive attributes. Moreover, secoiridoids are not only famous for their positive health impacts, but they are also responsible for the desired taste of olive oil [9]. Chemically based on the elenolic acid as the structural backbone [10], the major compounds of secoiridoids are aglycone forms of ligstroside aglycone (*p*–HPEA–EA; *p*–hydroxyphenyl–ethanol–elenolic acid) and oleuropein (3,4–DHPEA–EA; 3,4–dihydroxyphenylethanol–elenolic acid), as well as their chemically derived forms namely oleocanthal (*p*–HPEA–EDA; *p*–hydroxyphenyl–ethanol–elenolic acid dialdehyde) and oleacein (3,4–DHPEA–EDA; 3,4–dihydroxyphenylethanol–elenolic acid dialdehyde) [2,11]. As a member of the Oleaceae family, the olive is known to synthesize oleuropein and ligstroside [11]; however, different parts of the olive plant such as leaf, fruit, or oil include varying concentrations of phenolic compounds [12,13].

As a novel and greener technology, ultrasonic extraction has recently emerged by the increasing number of studies, which is a direct consequence of the increasing demand for energy-efficient, rapid, easy to use, and cheaper alternatives to conventional methods. In ultrasound technology, ultrasonic waves create microbubbles and result in an acoustic cavitation effect which enhances mass transfer mechanism from solid material to solvent phase due to disrupted cell structure, and better extraction yields can be obtained by tuning to the optimum sonication parameters [14,15]. Food applications of ultrasound technology spread in a wide range including mainly extraction of bioactive compounds [16,17,18] and lipid extraction [15,19], as well as other specific areas such as pigment extraction [20], non-thermal preservation and microbial inactivation [21,22], thawing of frozen foods [23], freezing of foods [24], pretreatment of drying of foods [25], and emulsification [26]. With regard to olives, although conventional extraction methods have been maintained and remain widely used, there has been a growing body of research investigating the application of ultrasound technology for the extraction of oil [27,28] and other bioactive compounds [29,30] from olive material.

From all these aspects, the goal of this study was to determine and evaluate the effects of ultrasound application on the composition of aforementioned bioactive compounds and selected physicochemical properties of olive extracts. Additionally, the ultrasonication conditions were assessed by using response surface methodology (RSM) and artificial neural network (ANN) to develop and compare mathematical models, thereby achieving a more accurate prediction of the experimental data. Furthermore, a multi-objective optimization (MOO) was performed to determine the optimal ultrasonication conditions by selecting the most appropriate responses to be maximized. The results of this study provided a practical overview for the olive industry to optimize ultrasonication-assisted extraction conditions for producing olive extracts with enhanced physicochemical characteristics and secoiridoid content by using ANN and RSM as useful tools for the prediction of the experimental data.

## 2. Materials and Methods

### 2.1. Materials

Olives belonging to the Sarı Ulak variety were obtained from local producers in the Mersin province of Türkiye. Fresh olive fruits were kept at a refrigeration temperature of 4 °C until the extractions and analyses. All chemicals and reagents used in the analyses including ethyl alcohol, methyl alcohol, phenolphthalein indicator, sodium hydroxide, Folin–Ciocalteu’s phenol reagent, sodium carbonate, and gallic acid standard were of analytical grade and purchased from various manufacturers including Supelco and Merck (Merck KGaA, Darmstadt, Germany).

### 2.2. Ultrasonic Extraction of Olives

The kernels of fresh olive fruits were removed manually, and then the remaining flesh was turned into olive paste using a spice grinder (KSPG–4812, Kiwi, Türkiye). A total of 10 ± 0.05 g of the pastes were weighed (XB 320M, Precisa Gravimetrics AG, Dietikon, Switzerland) in a glass beaker. After 50 mL of distilled water was introduced to the pastes, the glass beakers including the mixtures to be extracted were placed in the center of an ultrasonic bath (SK2210LHC, Kudos, Shanghai, China) operated under 53 kHz of ultrasonic frequency. Other sonication parameters were as follows: temperature (30, 40, 50 °C), time (5, 10, 15 min), ultrasonic power (60, 80, 100% of the maximum ultrasonication power of 90 W). Then, these parameters were introduced to the Box–Behnken design of experiment (DOE) as presented in Section 2.4. The extraction mixtures were then centrifuged at 10,000 rpm for 5 min (Z 326 K, Hermle Labortechnik GmbH, Wehingen, Germany). After centrifugation, the supernatants were stored in dark bottles at a refrigeration temperature of +4 °C.

### 2.3. Chemical Analyses

pH values of the samples were measured using a desktop pH meter (HI 2020 Edge, Hanna Instruments, Smithfield, RI, USA) after calibration on each day of analysis. Acidity by titration with alkali solution and the color index analyses of the samples were performed as described in the related methods [31].

#### 2.3.1. Total Phenolic Content (TPC) Analysis

Total phenolic content (TPC) analyses of the samples were conducted using Folin–Ciocalteu reagent as described in the method by Singleton et al. [32] with slight modifications. Briefly, the phenolics in the samples were extracted by a methanol:water mixture (1:1, *v*/*v*) and centrifuged at 7000 rpm for 5 min by using a refrigerated centrifuge (Z326 K, Hermle Labortechnik GmbH, Wehingen, Germany). Then, 1 mL of the extracts were mixed with 8.25 mL of water, 0.5 mL of Folin–Ciocalteu reagent, and 0.25 mL of sodium carbonate solution (20%, *w*/*v*), and the mixtures were kept in the dark for 30 min. The absorbance values of the samples were read at 760 nm wavelength in a UV–Vis spectrophotometer (V–730, Jasco Inc., Easton, MD, USA). The external calibration was performed using the calibration curve prepared with varying concentrations of gallic acid from 50 to 250 mg/kg. A good fit was obtained (R^2^ = 0.9982) from the calibration curve (Figure S1), and the results were expressed as mg gallic acid equivalent (GAE)/g extract.

#### 2.3.2. Determination of Secoiridoid Compositions

The compositions of the secoiridoids in terms of oleuropein, oleacein, and oleocanthal were determined by using an HPLC (Agilent 1260 Infinity, Agilent Technologies, Santa Clara, CA, USA). The column used in HPLC was a C–18 column (Thermo Scientific, Waltham, MA, USA) (150 mm length, 4.6 mm inner diameter, and 3 μm particle size). For analysis, the reference method was followed with slight modifications [33]. The injection conditions were as follows:

- Injection volume: 20 µL
- Wavelength: 280 nm
- Flow rate: 1 mL/min
- Oven temperature: 24 °C
- Mobile phase: (A, 60%) Distilled water acidified with 1% acetic acid; (B, %40) Methanol/Acetonitrile (1/1, *v*/*v*)

**Figure 1:**
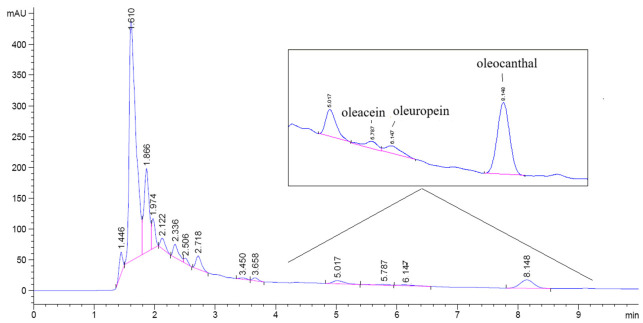
A sample chromatogram of selected secoiridoids in olive samples.

A sample chromatogram showing the peaks for the tested secoiridoids is given in Figure 1. According to Figure 1, the peaks for oleacein and oleuropein were very close to each other; thus, the potential influence of partial chromatographic overlap and limited signal-to-noise ratio on the quantification of low-abundance secoiridoids cannot be fully excluded and should be taken into consideration.

### 2.4. Design of Experiment (DOE) and Optimization

Ultrasonic extractions were conducted according to the Box–Behnken design of the response surface methodology (RSM). The selected three levels of the independent variables were as follows:

- $x_{1}$: 30, 40, 50 (°C; temperature)
- $x_{2}$: 5, 10, 15 (min; time)
- $x_{3}$: 60, 80, 100 (%; of maximum ultrasonic power)

All trials were conducted in duplicate, and the number of total trials was thirty including six trials in the center point. The experimental design including both coded and uncoded ultrasonic extraction variables is presented in Table 1. Data obtained from the experiments were used to build quadratic equations whose general structure is given in Equation (1).

(1) $$y_{i}={c_{0}+c}_{1}x_{1}+c_{2}x_{2}+c_{3}x_{3}+c_{11}x_{1}^{2}+c_{22}x_{2}^{2}+c_{33}x_{3}^{2}+c_{12}x_{1}x_{2}+c_{13}x_{1}x_{3}+c_{23}x_{2}x_{3}$$

where $y_{i}$ is the response; $c_{0}$ is constant; $c_{1}$, $c_{2}$, and $c_{3}$ are the linear coefficients; $c_{11}$,$\;c_{22}$, and $c_{33}$ are the quadratic coefficients; and $c_{12}$, $c_{13}$, and $c_{23}$ are the interaction coefficients.

The data from the experiments were analyzed using Minitab statistical software (Minitab 16, Minitab Inc., State College, PA, USA) within a confidence level of 95% in variance analysis (ANOVA). The determination of differences among the average values of each response was performed by Duncan’s multiple comparison test at 95% of confidence level. Also, 3D surface and contour plots were constructed to show the simultaneous binary effects of the process parameters using Statistica v10 package software (Statsoft Inc., Tulsa, OK, USA). The multi-objective optimization was performed using the built-in Response Optimizer of the Minitab software.

**Figure 2:**
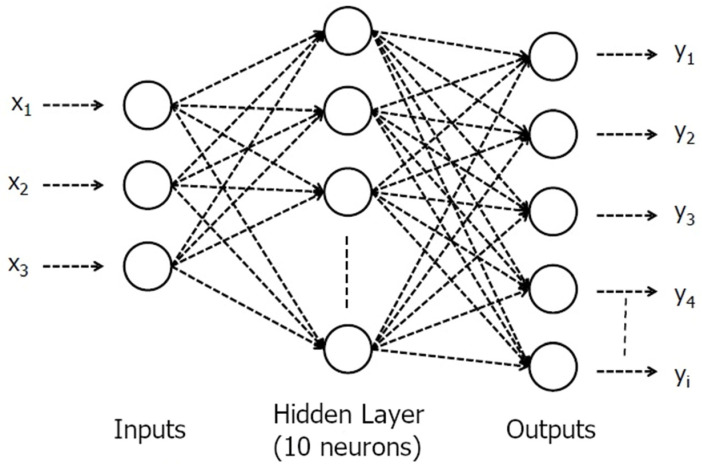
A schematic figure of artificial neural network (ANN).

An artificial neural network (ANN) was also utilized as an alternative approach in the estimation of responses from the mathematical models. MATLAB 2015a (Mathworks, Natick, MA, USA) was used to train the ANN using experimental data. Feed-forward backpropagation network type was used with one hidden layer including 10 neurons, and the network was trained using Levenberg–Marquardt function. The numbers of epoch and validation control were set to 1000 to achieve a better regression coefficient. A schematic figure of the applied ANN is given in Figure 2.

## 3. Results and Discussion

**Figure 3:**
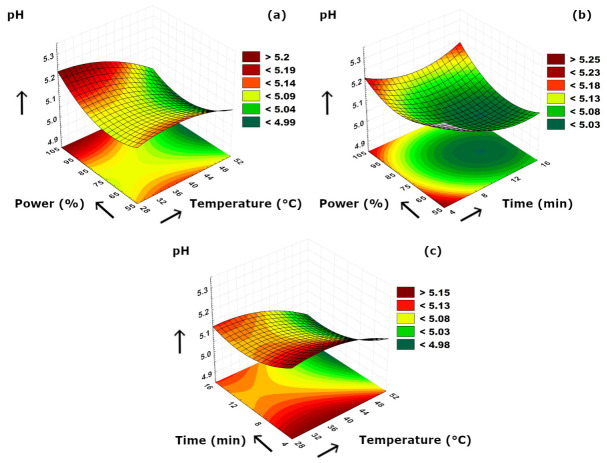
3D plot of pH versus binary ultrasonication parameters. (**a**) Power (%)-Temperature (°C). (**b**) Power (%)-Time (min). (**c**) Time (min)-Temperature (°C).

The experimental data obtained from pH, acidity, color index, TPC, and secoiridoid composition analyses are given in Table 2. The pH values of the samples were in a range of 4.94 ± 0.03 and 5.23 ± 0.12, as the highest and the lowest pH values were obtained at 40 °C–5 min–60% ultrasound power and 50 °C–15 min–80% ultrasound power conditions, respectively. The 3D plots of pH versus process parameters are given in Figure 3. According to Figure 3a, a decrease in pH values was observed with increasing temperature at a constant power level. This could be supported by organic acids in the sample which might tend to release and mix with the aqueous medium more at higher temperature levels. A similar phenomenon was also determined in power–time combination. It could be easily noticed from Figure 3b that decreasing ultrasonication duration at a constant power level also resulted in slightly higher pH values. At approximately 6 min of time level, increasing power level from 55 to 100% caused a slight decrease in pH at first; however, pH values tended to reach higher values up to 5.20 with a continuous increase in power. Figure 3c clearly revealed that increasing temperature resulted in lower pH values, as similar to Figure 3a in terms of more releasing of organic acids due to higher temperatures. On the contrary, longer extraction duration at a constant temperature caused a fluctuating trend in pH values as an initial decrease followed by a slight increment. This could be due to the following: (i) at first, cell walls broke apart due to ultrasound cavitation resulting in releasing more organic acids, (ii) then those organic acids might decompose at prolonged ultrasonication which caused the slight increase in pH values. In the literature, there are several studies reporting the ultrasonic extraction of olive material and the pH value relation. Most studies utilized pH value as a factor in RSM; however, some studies reported the optimal conditions including pH for their optimization studies. Rodríguez et al. [34] reported their optimal conditions which included pH as 5.6 to obtain the maximum extraction yield of polyphenols from olive pomace by using ultrasound. A similar study by Wang et al. [35] regarding the ultrasound-assisted extraction of polyphenols from olive leaves also indicated an optimum point for the maximization of polyphenols yield at which the optimal pH was 6.69. In another study by İlbay et al. [36], an optimum pH of 3.52 was suggested to maximize the TPC value when RSM was used in the ultrasound-assisted extraction of olive leaves. Trevisiol et al. [30] also reported a pH range of 6.58–7.14 for the ultrasound-assisted aqueous extracts of olive stones at different sonication periods. Our findings were in agreement with the published literature data.

According to Table 2, the acidity values of the olive extract samples varied between 0.548 and 0.557% (% oleic acid), as the highest acidity value was obtained at 40 °C–5 min–60% ultrasound power conditions. It could be stated that there was no statistically significant difference among the average acidity values of the samples. Moreover, it is conspicuous that the highest pH and acidity values were determined at the same extraction condition despite the well-known reverse relationship between acidity and pH. This could be explained by the presence of weak organic acids which increase titratable acidity without decreasing pH remarkably, as well as the potential buffering effect of extracted phenolic compounds which may resist changes in hydrogen ion concentration. The literature data mostly include acidity for olive oil and pH for olive aqueous extracts. In this study, we aimed to show the co-effects and simultaneous behavior of these two parameters to reveal the hidden positive correlation as stated above. Filipan et al. [37] reported that the free acidity values of ultrasound-pretreatment applied olive oil samples ranged between 0.19 and 0.42% oleic acid. In another study by Aydar et al. [38], the researchers investigated the effects of ultrasonication parameters on the extraction of olive oil. According to their results, the researchers managed to obtain an optimal acidity value of 0.24 mg oleic acid/100 g olive oil at their optimum point determined by RSM.

**Figure 4:**
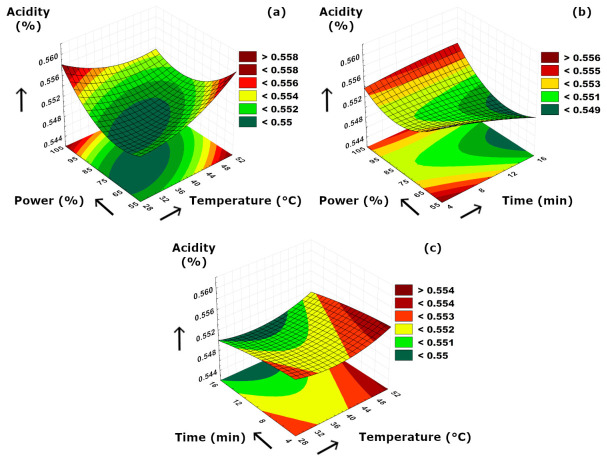
3D plot of acidity versus binary ultrasonication parameters. (**a**) Power (%)-Temperature (°C). (**b**) Power (%)-Time (min). (**c**) Time (min)-Temperature (°C).

The simultaneous interactions between the applied binary parameters versus acidity values are visualized in Figure 4. According to the figure, it could be concluded that individual increments in both temperature and power resulted in higher acidity values (Figure 4a), and the lowest acidity values were obtained at minimum levels of both temperature and ultrasound power. This could be due to the enhanced cavitation intensity and increased cell wall disruption at higher power and temperature levels, which led to higher amounts of organic or phenolic acids extracted and released to the medium. A similar augmentative effect of increased temperature on acidity was observed versus ultrasound application duration (Figure 4c). As can be seen from the figure, prolonged extraction time resulted in lower acidity values at constant power (Figure 4b) and constant temperature (Figure 4c) separately. This might be related to longer ultrasonication periods which would inevitably result in a possible degradation of organic acids. According to Figure 4b, increment in ultrasound power resulted in a fluctuated acidity value from a higher initial to lower amounts at moderate ultrasound power levels at a constant extraction time, which was related to an initial breakdown due to the starting cavitation effect resulting in higher acidity values while the acidic compounds started to decompose at increasing cavitation effect at moderate power levels.

The color values of the olive extract samples varied in a wide range between 20.46 and 83.70 in terms of photometric color index (PCI) (Table 2). PCI is a common spectrophotometric parameter used for the determination of color changes and color intensity in many liquid extracts such as beverages, oil, and plant extracts. This parameter includes four wavelengths of the electromagnetic spectrum corresponding to different color values, and the combined index (PCI) is beneficial in reflecting the concentrations of color-contributing compounds such as natural pigments, phenolic compounds, and their oxidized derivatives and Maillard reaction products. Since PCI is a sensitive spectrophotometric parameter, the observed differences in Table 2 might result from the combined effects of extraction conditions on pigment release, phenolic composition, and possible oxidation or degradation reactions. The differences in the average PCI values did not necessarily increase or decrease in a simple linear way with any individual variable; therefore, they should be considered as a whole parameter reflecting the complex behavior of the extraction mechanism. In olive products, the visible color is mainly formed by carotenoids and chlorophylls which have their characteristic absorbance wavelengths in the spectrophotometric analysis [39,40]. Although the number of studies regarding the PCI value of olive extracts are very limited, there are some studies reporting the PCI values of olive oil. In a recent study investigating the frying performance of safflower oil blended by refined olive pomace oil [41], researchers stated that the PCI values of refined olive pomace oil ranged between 5.84 and 7.73. In another study by Dolgun et al. [42], the researchers compared quality parameters of different olive oils obtained from different olive variety and also oils produced in two separate ways as organic and conventional methods. According to their findings, the PCI values of the olive oils ranged from 3.24 to 5.32, depending on the variety and production technique. Psathas et al. [43] investigated the effects of ripening stages on the quality of virgin olive oils, and they reported a wide range of PCI value from −59.93 to 21.40 at different stages of ripening. Depending on the nature of the olive extract, negative PCI values were also reported in olive oils. Giacomelli et al. [40] reported a mean PCI value of olive oil as −7.75 among other oil sources, and they stated that negative PCI value could be related to the higher concentration of chlorophylls that comes from the olive fruit itself. Trypidis et al. [44] also reported a negative range for their virgin olive oils samples from −9.5 to −68.3, and they also stated that this trend was in agreement with their total chlorophyll content.

**Figure 5:**
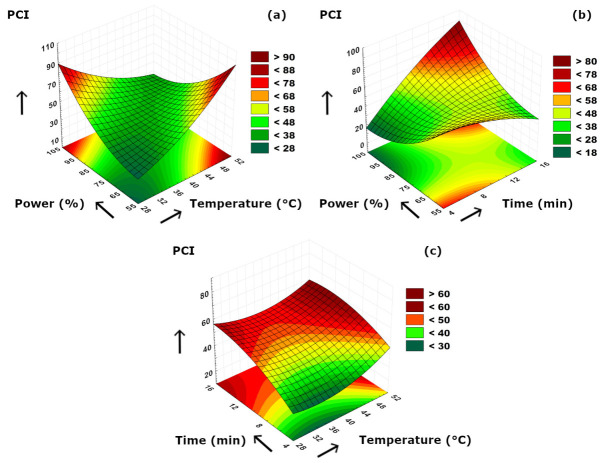
3D plot of PCI versus binary ultrasonication parameters. (**a**) Power (%)-Temperature (°C). (**b**) Power (%)-Time (min). (**c**) Time (min)-Temperature (°C).

The binary effects of the applied parameters on PCI are visualized in Figure 5. According to Figure 5a, it could be stated that PCI dramatically increased with either increasing temperature or ultrasound power. This might be related to the increased pigment release due to significantly disrupted cell walls due to power increment or the enhanced solubility of pigments at higher extraction temperatures. In contrast to the temperature, it could be concluded that shorter extraction duration led to increased PCI values when evaluated together with ultrasound power (Figure 5b). This phenomenon could be related to the degradation of chlorophyll and carotenoids or the oxidation of olive phenolics when longer extraction time was applied. In addition, the color pigments in olives are mostly unsaturated and thus, olive extract samples were prone to color loss at increased extraction periods during ultrasonication. According to Figure 5c, it was observed that time was the major influencing effect on PCI and changing temperatures at a constant, and shorter extraction duration was not significantly effective. At the beginning of the extraction period, the inadequate diffusion of color pigments to the extraction medium resulted in lower PCI values, and the cell structures of olives were not fully decomposed which caused a limited release of chromophoric compounds.

**Figure 6:**
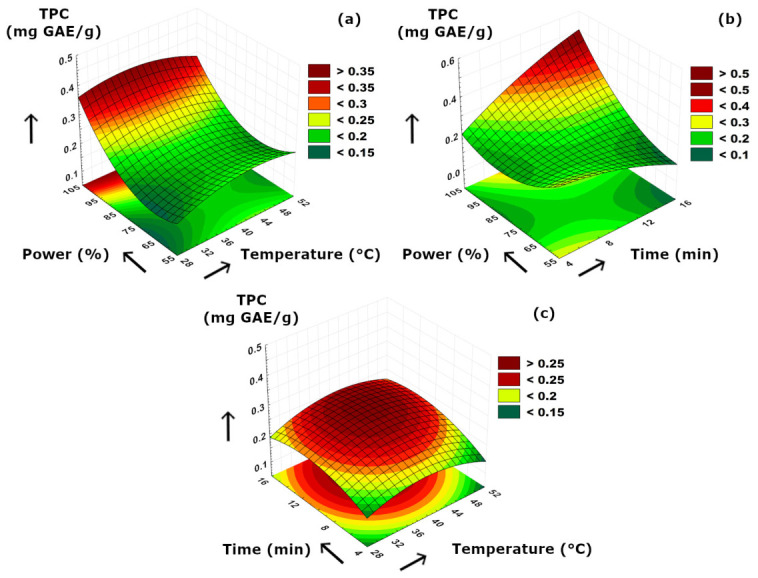
3D plot of TPC versus binary ultrasonication parameters. (**a**) Power (%)-Temperature (°C). (**b**) Power (%)-Time (min). (**c**) Time (min)-Temperature (°C).

The total phenolic contents (TPC) of the ultrasonically obtained olive extracts were in the range between 0.13 and 0.42 mg GAE/g, as the lowest and the highest TPCs were determined at 40 °C–15 min–60% ultrasound power and 40 °C–15 min–100% ultrasound power conditions, respectively. Our results presented in Table 2 suggested that TPC values of olive extract samples were highly dependent on extraction time. Although some previous studies concluded optimal shorter sonication duration for the best protection of phenolics [45,46], a clear positive effect of longer sonication periods on enhancing the TPC content of olive extracts was obvious in our case, which is visualized in Figure 6b. This was related with (i) possible hydrolysis of complex secoiridoids turning them into simpler phenolics at prolonged sonication, (ii) increased extractability of phenolics due to more solvent penetration, and (iii) breaking of possible phenolic–cell wall interactions resulting in higher phenolic concentration in free form, as well as improved release of aglycone forms. Figure 6a revealed that higher ultrasound power utilization had a significant effect on increasing TPC concentration specifically above 80% of power level. Since the effect of increasing temperature at a constant power level was limited, it could be stated that mass transfer of TPC was mainly dominated by ultrasound power in this binary comparison. According to the dome-shaped surface visible in Figure 6c, moderate temperature and time increment enabled a better release of TPCs and thus improved their mass transfer mechanism from the olive to the extraction medium.

Previous studies reveal that olive extracts have been thoroughly studied in terms of their total phenolic content and composition either extracted by using ultrasound or other techniques. Goldsmith et al. [47] reported the phenolic content of aqueous ultrasonic-extracted olive pomace under an RSM design. Their actual and maximized TPC values were 19.71 and 22.02 mg GAE/g when the ultrasonication was utilized, while a lower TPC of 13.76 mg GAE/g was obtained when no ultrasound was used. The study also emphasized that the use of ultrasonication enabled a better extraction making the water have a greater extraction efficiency, and higher TPC values were obtained. According to another study by Trevisiol et al. [30], the TPC of aqueous extracts obtained by ultrasonication from olive stones ranged from 247.5 to 367.3 mg GAE/g dry weight when the sonication time varied from 0 (no ultrasonication) to 120 min. Kim et al. [48] also reported the total phenolic contents of aqueous extracts of olive pomace obtained by ultrasound as 24.43, 24.69, 20.98, and 14.14 mg GAE/g at their corresponding sample to solvent ratios as 1, 2, 4, and 8 dry olive pomace per 20 mL of control sample. As another instance regarding olive oil, Peres et al. [49] studied the ultrasound-assisted coextraction of olive and thyme and they reported that the phenolic contents of olive oil samples extracted without and with ultrasonication were 264.22 and 325.15 mg GAE/kg oil. This increment corresponded to a 23% improvement in the phenolic content when ultrasound was utilized. It could be concluded that our findings from this study aligned with the previously published data.

Regarding secoiridoid contents, the concentrations of oleuropein, oleacein, and oleocanthal ranged between 5.33 and 34.39 ng/µL, 0.76 and 6.03 ng/µL, and 3.77 and 14.16 ng/µL, respectively (Table 2). For a better comparison with published data, these ranges can be also converted and thus, respectively, corresponded to 26.65–171.95 mg/kg, 3.8–30.15 mg/kg, and 18.85–70.8 mg/kg when the amount of olive sample and the extraction volume are taken into consideration. It could be concluded that our findings clearly align with several published data reporting the secoiridoids ranges either in olive fruit or olive oil [50,51,52]. As can be seen from Table 2, the oleuropein concentrations were higher than oleacein and oleocanthal. Previous reports in the literature also indicate that oleuropein is abundant in olive fruit or leaves [5,12,53], while oleacein and oleocanthal are the distinctive secoiridoids of olive oil [9,54]. Also, it should be noted that the concentrations of these secoiridoids may vary depending on variety, seasonal changes, and environmental factors. According to Filipan et al. [37], the oleuropein aglycone, oleacein, and oleocanthal contents of virgin olive oils obtained from ultrasonic-treated olive pastes belonging to different Croatian olive varieties ranged between 24 and 110 mg/kg, 38 and 145 mg/kg, and 42 and 94 mg/kg, respectively. Servili et al. [55] applied low-frequency high-power ultrasound treatment to olive pastes. Their range for oleacein was between 3.9 and 467.2 mg/kg for the Arbequina sample treated with ultrasound at 1.7 bar and for Coratina sample treated with ultrasound at 3.5 bar, respectively. Similarly, their oleocanthal concentrations were in a wide range of 5.5–156.8 mg/kg. According to Khanlar et al. [56], a maximum oleuropein extraction yield of 0.024 mg oleuropein/g olive leaf d.m could be obtained when moderate electric field (MEF) and power ultrasound (US) were combined, and also a significant reduction of 75% in the extraction duration was achieved successfully.

**Figure 7:**
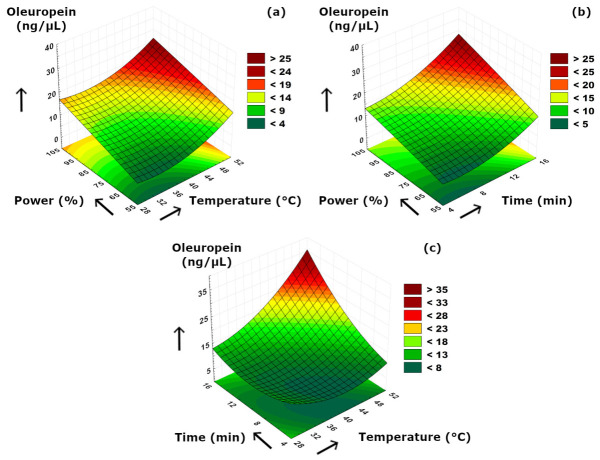
3D plot of oleuropein versus binary ultrasonication parameters. (**a**) Power (%)-Temperature (°C). (**b**) Power (%)-Time (min). (**c**) Time (min)-Temperature (°C).

**Figure 8:**
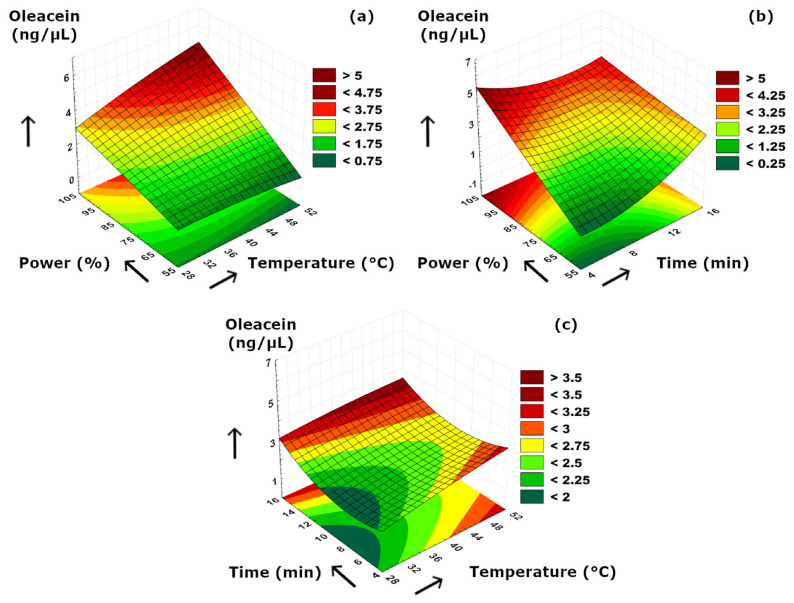
3D plot of oleacein versus binary ultrasonication parameters. (**a**) Power (%)-Temperature (°C). (**b**) Power (%)-Time (min). (**c**) Time (min)-Temperature (°C).

**Figure 9:**
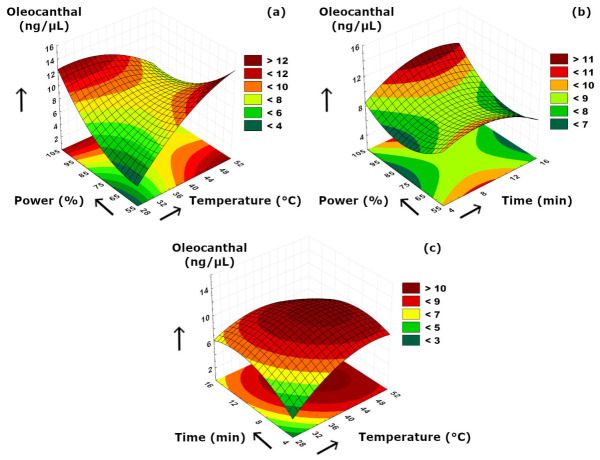
3D plot of oleocanthal versus binary ultrasonication parameters. (**a**) Power (%)-Temperature (°C). (**b**) Power (%)-Time (min). (**c**) Time (min)-Temperature (°C).

The effects of sonication parameters on the secoiridoids compositions are visualized in Figure 7, Figure 8 and Figure 9. Figure 7a and Figure 8a suggested that power–temperature combination had a similar effect on both oleuropein and oleacein content. The concentrations of both compounds strongly increased with higher cavitation effect due to increased power. In Figure 7a, it is also clearly visible that increasing temperature had a positive effect on improving the solubility and diffusion of oleuropein, and thus, resulting in higher concentrations. A similar but less significant observation can also be suggested for oleacein concentration as seen in Figure 8a. The trend of concentration change for oleocanthal was much different than other two secoiridoids. We should note that, although increasing ultrasonication time may enhance the release of phenolic compounds from the plant matrix, prolonged exposure may also promote partial degradation or transformation of sensitive secoiridoid compounds such as oleocanthal; therefore, a longer extraction time does not necessarily result in a higher oleocanthal concentration, as can be also presented in Table 2. Similarly, at elevated temperature values, such reactions may partially diminish the extraction yield by reduced compound stability under more intense processing conditions. According to Figure 9a, the ultrasound power and temperature had a strong synergistic effect on the oleocanthal concentration. Increasing both factors either individually or simultaneously provided a high oleocanthal concentration. Slight decreases in the oleocanthal concentration at low and moderate power levels occurred at prolonged extraction periods (Figure 9b) which might be related to the reactive aldehyde groups present in oleocanthal that are prone to oxidation reaction. The curved shape of the response surface presented in Figure 9c suggested that higher temperatures might cause thermal degradation of oleocanthal, while longer extraction time was also detrimental in terms of oxidation as similar to Figure 9b. We should also note that, although oleuropein, oleacein, and oleocanthal are structurally related secoiridoid compounds, the current experimental design does not allow direct evaluation of their possible interconversion; thus, mutual transformation among these components was not confirmed in the present study.

### Results of Statistical Evaluation, Mathematical Modeling and Optimization

The results of variance analysis (ANOVA) from olive extracts obtained by ultrasound are presented in Table 3. According to the table, *p* values of regression models for all responses except pH and acidity were below the confidence level (0.05) which meant that the models for these responses were statistically meaningful and could be safely used for the evaluation of experimental data. The reason for the models being statistically insignificant for pH and acidity was the narrow range of experimental data for these analyses. The data obtained from different sonication trials for pH and acidity analyses were not significantly affected by ultrasonication parameters, and thus it could be stated that creating the best model was not the primary goal for these analyses. The lack of fit (LoF) values for pH, acidity, PCI, and TPC were statistically insignificant (*p* > 0.05), resulting in a fine conclusion that the experimental data and the mathematical model closely fit to each other, and the models could be used without hesitation in the presentation of experimental data. However, LoF values were statistically significant for oleuropein, oleocanthal, and oleacein, indicating that more improvements should be made on the models in terms of adding more terms or changing design size. Since the regression models were applicable (*p* < 0.05) for these secoiridoids, changing the number of experiments must be the key solutions instead of changing models unnecessarily. Regarding individual effects, TPC, oleuropein, and oleocanthal were significantly affected by ultrasound power, time, and temperature, respectively.

Regarding modeling, the experimental findings from the analyses were used to create quadratic mathematical models by using RSM, and these models are listed in Table 4. Subsequently, these models were incorporated for a multi-objective optimization (MOO) procedure where the selected attributes such as TPC, oleuropein, oleacein, and oleocanthal concentrations were aimed to maximize. Since the experimental findings for pH, acidity, and PCI ranged in a narrow region, they were not chosen for MOO. The weights of the selected responses in MOO were chosen arbitrarily to obtain the best desirability values, where the weight of TPC was 0.4, and the weights of the each secoiridoid concentration were equally set to 0.2. The parameters used in the MOO setup are tabulated in Table 5.

The lower, target, and upper limits of the responses were determined according to the experimental data range. The starting points of the temperature, time, and ultrasound power were 30 °C, 5 min, and 60%, respectively. After performing the MOO, the optimal conditions for reaching the maximum of the selected responses were determined as extraction temperature of 43.13 °C, extraction time of 15 min, and ultrasound power of 100%. The predicted responses for the selected parameters at this optimal point were obtained as follows:

- TPC = 0.4252 mg GAE/g [desirability: 0.9373]
- Oleuropein = 26.2234 ng/μL [desirability: 1.0000]
- Oleacein = 3.8640 ng/μL [desirability: 0.8880]
- Oleocantha l= 10.0118 ng/μL [desirability: 1.0000]

The overall desirability of this process was determined as 0.955, which can be concluded as this optimization step could be considered as successful for an approximate percent of 95.5%.

**Figure 10:**
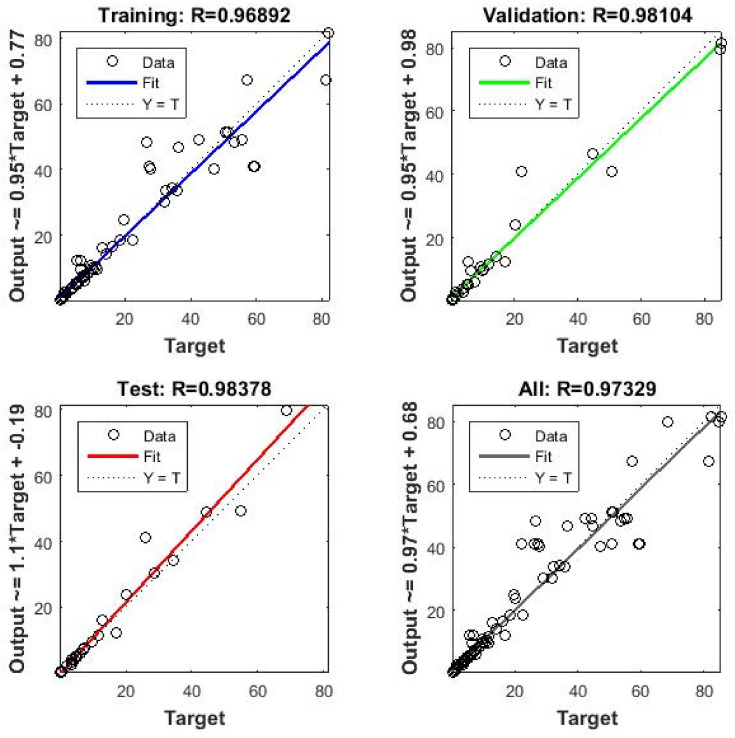
The regression values of the ANN.

In the next step, the responses from each ultrasonic extraction conditions were also estimated by using RSM and ANN, as described in Section 2.4. In the ANN step, three input parameters (temperature, time, and ultrasound power) were processed in a hidden layer including 10 neurons inside, and seven responses were estimated after the training of the ANN was completed when an acceptable regression coefficient (R) was achieved. The R value is the key indicator of a successful training session of ANN enabling the model to estimate the responses as close as possible to the experimental data. In this study, the regressions for training, validation, test, and overall process were 0.969, 0.981, 0.984, and 0.973, respectively (Figure 10).

After the responses of all analyses in all ultrasonication trials were estimated by ANN which passed through several training runs, a further comparison was performed by RSM to better clarify the prediction performances of the two models. The averages of experimental data and the predicted responses from both RSM and ANN are tabulated in Table 6. In some of the cases, RSM was more beneficial in the better prediction of data, while the predictions of ANN were more identical to the experimental data in specific circumstances. For some instances from the table, the average of experimental pH value (5.225) at 40 °C–5 min–60% of ultrasound power conditions was predicted by RSM and ANN as 5.198 and 5.193, respectively. Regarding another response, TPC obtained in the extraction at 40 °C–10 min–80%, the average TPC value of 0.224 mg GAE/g extract was predicted as 0.224 and 0.242 by RSM and ANN with their corresponding differences from experimental data as 0 and 0.018, respectively. As is clearly evident in most cases, the differences of RSM and ANN from experimental data can be neglected, which makes these approaches safely usable in the prediction step. Among all data placed in Table 6, the highest difference of 8.359 (Δ_(ANN–EXP)_) belonged to the color index of the sample extracted at 50 °C–10 min–100% ultrasound power conditions. The findings from the Table 6 indicated that TPC and secoiridoid compositions may be considered as the most promising and significant parameters for the practical application of the present study, as they are the key parameters indicating the bioactive composition and potential functional value of the olive extracts; however, pH, acidity, and PCI are generally considered as supportive physicochemical quality indicators.

## 4. Conclusions

The findings of this study revealed that pH values of the aqueous olive extracts obtained in ultrasound extraction ranged between 4.94 and 5.23. The changes in pH value occurred in a narrow range, and no significant difference was determined among the samples. Increased releasing of organic acids was thought to be the main reason for pH decrease at higher extraction temperatures. The titratable acidity values of the olive extract samples were analyzed between 0.548 and 0.557% (% oleic acid). As similar to pH values, no statistically significant difference was determined among the average acidity values of the samples. The simultaneous increase in pH and acidity was originated from the presence of weak organic acids causing the increase in titratable acidity without decreasing pH. Regarding color, the PCI values of the samples ranged between 20.46 and 83.70. The increased ultrasound power and temperature levels resulted in more releasing of coloring pigments such as carotenoids and chlorophyll to the aqueous mixture which led to higher PCI values. Oppositely, lower PCI values were obtained due to possible oxidation of these pigments at prolonged ultrasound extractions. As the duration of the extraction increased, the TPC values of the samples increased which might be related to such factors as the possible hydrolysis of complex secoiridoids, the increased extractability of phenolics, or the breaking of possible phenolic–cell wall interactions. The concentrations of oleuropein and oleacein had a positive correlation with higher ultrasound power due to higher cavitation effect. Oleuropein concentration was mostly affected by higher temperatures as the solubility and diffusion of oleuropein increased at higher temperatures. The quadratic models were found significant for all responses except pH and acidity, and thus, concluded to be applicable for the presentation of experimental data successfully. The optimum extraction condition to obtain the maximum TPC, oleuropein, oleacein, and oleocanthal concentrations was the extraction temperature of 43.13 °C, extraction time of 15 min, and ultrasound power of 100%. The overall desirability of the multi-objective optimization was 0.955. The comparison between RSM and ANN in the prediction of experimental data revealed that both methods had predominance in particular extraction runs. The overall findings of this study concluded that olive extracts could be successfully obtained by ultrasonication, and the use of both ANN and RSM could make the challenging experimental procedure much easier in a reliable, reproducible, and rapid approach with a solid statistical approach. Moreover, the ANN approach made a significant contribution to the present study by effectively modeling the complex relationships between ultrasonication parameters and the selected physicochemical properties of olive extracts, making ANN a powerful tool for prediction and process optimization. Future studies should focus on validating the developed ANN and RSM models as well as developing new models under different processing conditions and evaluate the possibility of scaling up of the optimized extraction process for industrial applications including the olive industry.

## Figures and Tables

**Table 1:** Ultrasonic extraction conditions of Box–Behnken design.

Trials	Coded Variables			Uncoded Variables		
	*x*_1_	*x*_2_	*x*_3_	*x*_1_ (°C)	*x*_2_ (min)	*x*_3_ (%)
1	0	-	-	40	5	60
2	-	0	+	30	10	100
3	0	+	+	40	15	100
4	+	0	+	50	10	100
5	0	0	0	40	10	80
6	-	0	+	30	10	100
7	0	0	0	40	10	80
8	0	+	-	40	15	60
9	+	+	0	50	15	80
10	-	-	0	30	5	80
11	0	-	+	40	5	100
12	-	0	-	30	10	60
13	0	0	0	40	10	80
14	-	-	0	30	5	80
15	0	+	+	40	15	100
16	-	+	0	30	15	80
17	0	-	-	40	5	60
18	+	-	0	50	5	80
19	+	0	+	50	10	100
20	-	+	0	30	15	80
21	+	0	-	50	10	60
22	+	-	0	50	5	80
23	0	0	0	40	10	80
24	0	-	+	40	5	100
25	-	0	-	30	10	60
26	+	0	-	50	10	60
27	+	+	0	50	15	80
28	0	+	-	40	15	60
29	0	0	0	40	10	80
30	0	0	0	40	10	80

**Table 2:** Values of pH, acidity, color index, TPC, and secoiridoid compositions.

Ultrasonic Extraction Conditions			pH	Acidity (%)	Photometric Color Index (PCI)	Total Phenolic Content (TPC) (mg GAE/g) (*Values in Parentheses Are Equal to mg GAE/mL*)	Secoiridoid Composition (ng/µL) (*Values in Parentheses Are Equal to mg/mL*)		
***x*** **_1_** **(°C)**	*x*_2_ (min)	*x*_3_ (%)					Oleuropein	Oleacein	Oleocanthal
40	5	60	5.23 ^A^ ± 0.12	0.557 ^A^ ± 0.004	50.95 ^BCD^ ± 0.50	0.26 ^BCD^ ± 0.04 (0.013)	6.71 ^FG^ ± 1.67 (0.00671)	0.76 ^F^ ± 0.07 (0.00076)	8.62 ^BCD^ ± 0.03 (0.00862)
30	10	100	5.17 ^AB^ ±0.02	0.556 ^A^ ± 0.004	69.15 ^ABC^ ± 17.10	0.38 ^AB^ ± 0.12 (0.019)	16.08 ^BCD^ ± 0.23 (0.01608)	2.19 ^DE^ ± 0.50 (0.00219)	9.88 ^BC^ ± 0.23 (0.00988)
40	15	100	5.12 ^AB^ ± 0.09	0.549 ^A^ ± 0.001	76.71 ^AB^ ± 11.45	0.42 ^A^ ± 0.07 (0.021)	20.18 ^B^ ± 0.67 (0.02018)	3.60 ^BC^ ± 0.01 (0.0036)	11.65 ^AB^ ± 0.07 (0.01165)
50	10	100	5.09 ^AB^ ± 0.17	0.554 ^A^ ± 0.002	39.93 ^CD^ ± 19.11	0.31 ^ABC^ ± 0.06 (0.016)	19.69 ^BC^ ± 0.03 (0.01969)	3.28 ^BCD^ ± 0.07 (0.00328)	7.42 ^CD^ ± 0.06 (0.00742)
40	15	60	5.11 ^AB^ ± 0.16	0.548 ^A^ ± 0.002	37.49 ^D^ ± 13.77	0.13 ^D^ ± 0.04 (0.007)	8.64 ^EFG^ ± 1.02 (0.00864)	0.99 ^EF^ ± 0.06 (0.00099)	6.10 ^DE^ ± 0.13 (0.0061)
50	15	80	4.94 ^B^ ± 0.03	0.548 ^A^ ± 0.005	48.70 ^BCD^ ± 8.87	0.22 ^CD^ ± 0.04 (0.011)	34.39 ^A^ ± 0.47 (0.03439)	4.27 ^B^ ± 0.10 (0.00427)	7.14 ^CD^ ± 0.07 (0.00714)
30	5	80	5.18 ^A^ ± 0.17	0.552 ^A^ ± 0.003	34.06 ^D^ ± 2.48	0.14 ^D^ ± 0.01 (0.007)	5.33 ^G^ ± 0.45 (0.00533)	1.01 ^EF^ ± 0.13 (0.00101)	3.77 ^E^ ± 0.01 (0.00377)
40	5	100	5.14 ^AB^ ± 0.01	0.549 ^A^ ± 0.004	20.46 ^D^ ± 2.87	0.22 ^CD^ ± 0.00 (0.011)	14.11 ^BCDE^ ± 0.47 (0.01411)	6.03 ^A^ ± 0.51 (0.00603)	11.09 ^B^ ± 0.42 (0.01109)
30	10	60	5.03 ^AB^ ± 0.01	0.553 ^A^ ± 0.000	30.39 ^D^ ± 2.25	0.18 ^CD^ ± 0.01 (0.009)	6.20 ^FG^ ± 0.30 (0.0062)	2.12 ^DE^ ± 0.47 (0.00212)	6.16 ^DE^ ± 0.01 (0.00616)
30	15	80	5.12 ^AB^ ± 0.01	0.551 ^A^ ± 0.002	50.01 ^BCD^ ± 7.82	0.19 ^CD^ ± 0.01 (0.01)	12.97 ^CDEF^ ± 0.57 (0.01297)	3.49 ^BCD^ ± 0.01 (0.00349)	7.58 ^CD^ ± 0.19 (0.00758)
50	5	80	5.05 ^AB^ ± 0.04	0.550 ^A^ ± 0.001	40.55 ^CD^ ± 5.78	0.15 ^D^ ± 0.00 (0.008)	9.44 ^DEFG^ ± 0.74 (0.00944)	2.30 ^CDE^ ± 0.07 (0.0023)	7.51 ^CD^ ± 0.01 (0.00751)
50	10	60	5.05 ^AB^ ± 0.04	0.555 ^A^ ± 0.004	83.70 ^A^ ± 2.22	0.20 ^CD^ ± 0.00 (0.01)	10.35 ^DEFG^ ± 0.73 (0.01035)	1.51 ^EF^ ± 0.01 (0.00151)	14.16 ^A^ ± 0.13 (0.01416)
40	10	80	5.06 ^AB^ ± 0.10	0.548 ^A^ ± 0.006	40.87 ^CD^ ± 17.48	0.22 ^CD^ ± 0.08 (0.011)	9.70 ^DEFG^ ± 2.17 (0.0097)	2.30 ^CDE^ ± 0.22 (0.0023)	9.26 ^BCD^ ± 0.167 (0.00926)

*Results are expressed in terms of average ± standard deviations. The differences between the average data having different letters within the same columns are statistically significant (p < 0.05).*

**Table 3:** Variance analysis (ANOVA) results of olive extracts.

Response	pH		Acidity		Color Index (PCI)		Total Phenolic Content (TPC)		Oleuropein		Oleacein		Oleocanthal	
Source	F	*p*	F	*p*	F	*p*	F	*p*	F	*p*	F	*p*	F	*p*
*Model*	1.24	0.325	0.80	0.617	6.19	0.000	5.84	0.001	7.52	0.000	2.81	0.026	4.58	0.002
$x_{1}$	0.74	0.401	0.03	0.875	1.11	0.305	1.95	0.178	4.06	0.057	0.03	0.858	15.53	0.001
$x_{2}$	1.11	0.305	0.34	0.564	1.73	0.203	2.73	0.114	5.82	0.026	0.23	0.636	2.20	0.153
$x_{3}$	1.13	0.301	0.93	0.345	0.35	0.563	7.24	0.014	0.02	0.883	0.03	0.868	0.16	0.691
${{(x}_{1})}^{2}$	0.52	0.478	0.73	0.402	1.77	0.198	1.79	0.196	3.97	0.060	0.02	0.902	3.95	0.061
${{(x}_{2})}^{2}$	1.28	0.271	0.00	0.959	0.60	0.446	1.28	0.271	2.50	0.129	1.48	0.237	4.19	0.054
${{(x}_{3})}^{2}$	1.99	0.173	2.46	0.133	4.09	0.057	7.68	0.012	0.01	0.938	0.00	0.954	4.86	0.039
$x_{1}\times x_{2}$	0.11	0.746	0.00	0.968	0.21	0.653	0.09	0.765	7.62	0.012	0.10	0.758	2.53	0.127
$x_{1}\times x_{3}$	0.48	0.497	1.73	0.203	23.27	0.000	0.39	0.540	0.01	0.932	1.09	0.310	15.86	0.001
$x_{2}\times x_{3}$	0.53	0.475	0.75	0.398	16.61	0.001	16.65	0.001	0.44	0.517	2.66	0.119	1.38	0.255
*Lack-of-Fit (LoF)*	1.23	0.328	2.00	0.152	0.47	0.708	0.11	0.950	7.99	0.002	20.33	0.000	7.38	0.002

**Table 4:** Mathematical models of the responses from RSM *.

Responses	Models **
*pH*	$=5.54542+0.02792x_{1}-0.05096x_{2}-0.01726x_{3}-0.00026x_{1}^{2}+0.00162x_{2}^{2}+0.00013x_{3}^{2}-0.00023x_{1}x_{2}\;\;\;\;\;\;\;\;\;\;\;\;\;\;\;\;\;\;\;\;\;\;\;\;\;\;\;-0.00012x_{1}x_{3}+0.00025x_{2}x_{3}$
*Acidity*	$=0.586917-0.000230x_{1}-0.001258x_{2}-0.000696x_{3}+0.000014x_{1}^{2}-0.000003x_{2}^{2}+0.000006x_{3}^{2}\;\;\;\;\;\;\;\;\;\;\;\;\;\;\;\;\;\;\;\;\;\;\;\;\;\;\;+0.000001x_{1}x_{2}-0.000010x_{1}x_{3}+0.000013x_{2}x_{3}$
*PCI*	$=26.3428+4.2670x_{1}-7.9413x_{2}-1.1931x_{3}+0.0593x_{1}^{2}-0.1385x_{2}^{2}+0.0225x_{3}^{2}-0.0390x_{1}x_{2}\;\;\;\;\;\;\;\;\;\;\;\;\;\;\;\;\;\;\;\;\;\;\;\;\;\;\;-0.1032x_{1}x_{3}+0.1743x_{2}x_{3}$
*TPC*	$=0.787333+0.026548x_{1}-0.046738x_{2}-0.025548x_{3}-0.000279x_{1}^{2}-0.000945x_{2}^{2}+0.000144x_{3}^{2}\;\;\;\;\;\;\;\;\;\;\;\;\;\;\;\;\;\;\;\;\;\;\;\;\;\;\;+0.000121x_{1}x_{2}-0.000062x_{1}x_{3}+0.000818x_{2}x_{3}$
*Oleuropein*	$=68.9725-2.9964x_{1}-5.3403x_{2}+0.1105x_{3}+0.0325x_{1}^{2}+0.1032x_{2}^{2}+0.0003x_{3}^{2}+0.0866x_{1}x_{2}\;\;\;\;\;\;\;\;\;\;\;\;\;\;\;\;\;\;\;\;\;\;\;\;\;\;\;-0.0007x_{1}x_{3}+0.0103x_{2}x_{3}$
*Oleacein*	$=-2.31917-0.07029x_{1}+0.27658x_{2}+0.03242x_{3}-0.00053x_{1}^{2}+0.02068x_{2}^{2}+0.00006x_{3}^{2}-0.00255x_{1}x_{2}\;\;\;\;\;\;\;\;\;\;\;\;\;\;\;\;\;\;\;\;\;\;\;\;\;\;\;+0.00212x_{1}x_{3}-0.00665x_{2}x_{3}$
*Oleocanthal*	$=-45.2933+2.4522x_{1}+1.3757x_{2}-0.1254x_{3}-0.0136x_{1}^{2}-0.0559x_{2}^{2}+0.0038x_{3}^{2}-0.0209x_{1}x_{2}\;\;\;\;\;\;\;\;\;\;\;\;\;\;\;\;\;\;\;\;\;\;\;\;\;\;\;-0.0131x_{1}x_{3}+0.0077x_{2}x_{3}$

* Uncoded factors were used in the quadratic models; ** x_1_ (Temperature, °C), x_2_ (Time, min), x_3_ (Power, %).

**Table 5:** Multi-objective optimization (MOO) setup used in RSM.

Parameters	Goal	Lower	Target	Upper	Weight
TPC (mg GAE/g)	Maximize	0	0.5	0.5	0.4
Oleuropein (ng/μL)	Maximize	5	20.0	20.0	0.2
Oleacein (ng/μL)	Maximize	0	7.0	7.0	0.2
Oleocanthal (ng/μL)	Maximize	6	10.0	10.0	0.2

**Table 6:** Comparison between the experimental data and the predicted responses from RSM and ANN.

Ultrasound Parameters			Responses						
x_1_	x_2_	x_3_	pH	Acidity (%)	PCI	Total Phenolic Content–TPC (mg GAE/g)	Oleuropein (ng/μL)	Oleacein (ng/μL)	Oleocanthal (ng/μL)
**40**	**5**	**60**							
*Average experimental data (EXP)*			5.225	0.557	50.954	0.261	6.710	0.760	8.620
*Prediction by RSM*			5.198	0.554	55.005	0.252	3.601	0.684	9.325
*Prediction by ANN*			5.193	0.545	51.350	0.262	6.733	0.769	8.248
Δ*_(RSM–EXP)_*			−**0.027**	−**0.003**	4.052	−0.009	−3.109	−0.076	0.705
Δ*_(ANN–EXP)_*			−0.032	−0.012	**0.397**	**0.001**	**0.023**	**0.009**	**−0.372**
**30**	**10**	**100**							
*Average experimental data (EXP)*			5.165	0.556	69.149	0.337	16.080	2.190	9.880
*Prediction by RSM*			5.163	0.555	73.229	0.328	13.824	2.746	11.540
*Prediction by ANN*			5.183	0.557	67.375	0.324	16.624	2.319	10.047
Δ*_(RSM–EXP)_*			**−0.002**	−0.001	4.080	**−0.009**	−2.256	0.556	1.660
Δ*_(ANN–EXP)_*			0.018	0.001	**−1.774**	−0.013	**0.544**	**0.129**	**0.167**
**40**	**15**	**100**							
*Average experimental data (EXP)*			5.120	0.549	76.710	0.420	20.180	3.600	11.650
*Prediction by RSM*			5.148	0.553	72.658	0.429	23.289	3.676	10.945
*Prediction by ANN*			5.110	0.550	79.621	0.404	23.914	4.128	11.561
Δ*_(RSM–EXP)_*			0.027	0.003	−4.052	**0.009**	**3.109**	**0.076**	−0.705
Δ*_(ANN–EXP)_*			**−0.010**	**0.001**	**2.911**	−0.015	3.734	0.528	**−0.089**
**50**	**10**	**100**							
*Average experimental data (EXP)*			5.090	0.554	39.933	0.308	19.690	3.280	7.420
*Prediction by RSM*			5.026	0.552	39.285	0.311	21.876	4.234	8.520
*Prediction by ANN*			5.001	0.546	48.292	0.364	24.820	3.261	7.531
Δ*_(RSM–EXP)_*			**−0.064**	**−0.001**	**−0.648**	**0.003**	**2.186**	0.954	1.100
Δ*_(ANN–EXP)_*			−0.089	−0.007	8.359	0.055	5.130	**−0.019**	**0.111**
**40**	**15**	**60**							
*Average experimental data (EXP)*			5.110	0.548	37.486	0.133	8.640	0.990	6.100
*Prediction by RSM*			5.071	0.549	36.866	0.135	11.679	2.576	8.155
*Prediction by ANN*			5.040	0.554	40.168	0.127	8.510	0.946	6.063
Δ*_(RSM–EXP)_*			**−0.039**	**0.001**	**−0.619**	**0.002**	3.039	1.586	2.055
Δ*_(ANN–EXP)_*			−0.070	0.007	2.683	−0.006	**−0.130**	**−0.044**	**−0.037**
**50**	**15**	**80**							
*Average experimental data (EXP)*			4.940	0.552	48.700	0.218	34.390	4.270	7.140
*Prediction by RSM*			4.977	0.550	53.400	0.207	29.095	3.240	6.745
*Prediction by ANN*			4.919	0.548	49.136	0.206	34.377	4.270	7.245
Δ*_(RSM–EXP)_*			0.037	**−0.002**	4.700	**−0.012**	−5.295	−1.030	−0.395
Δ*_(ANN–EXP)_*			**−0.021**	−0.004	**0.436**	−0.013	**−0.013**	**0.000**	**0.105**
**30**	**5**	**80**							
*Average experimental data (EXP)*			5.180	0.549	34.056	0.140	5.330	1.010	3.770
*Prediction by RSM*			5.143	0.551	29.357	0.152	10.625	2.040	4.165
*Prediction by ANN*			5.291	0.546	33.750	0.187	5.331	1.049	3.772
Δ*_(RSM–EXP)_*			**−0.037**	**0.002**	−4.700	**0.012**	5.295	1.030	0.395
Δ*_(ANN–EXP)_*			0.111	−0.004	**−0.306**	0.047	**0.001**	**0.039**	**0.002**
**40**	**5**	**100**							
*Average experimental data (EXP)*			5.135	0.553	20.456	0.221	14.110	6.030	11.090
*Prediction by RSM*			5.174	0.552	21.075	0.219	11.071	4.444	9.035
*Prediction by ANN*			5.107	0.553	18.502	0.197	14.150	6.030	10.315
Δ*_(RSM–EXP)_*			0.039	−0.001	**0.619**	**−0.002**	−3.039	−1.586	−2.055
Δ*_(ANN–EXP)_*			**−0.028**	**0.000**	−1.953	−0.024	**0.040**	**0.000**	**−0.775**
**30**	**10**	**60**							
*Average experimental data (EXP)*			5.025	0.549	30.385	0.175	6.200	2.120	6.160
*Prediction by RSM*			5.089	0.550	31.033	0.173	4.014	1.166	5.060
*Prediction by ANN*			5.065	0.549	30.219	0.157	5.963	2.160	6.110
Δ*_(RSM–EXP)_*			0.064	0.001	0.648	**−0.003**	−2.186	−0.954	−1.100
Δ*_(ANN–EXP)_*			**0.040**	**0.000**	**−0.166**	−0.018	**−0.237**	**0.040**	**−0.050**
**30**	**15**	**80**							
*Average experimental data (EXP)*			5.115	0.551	50.010	0.185	12.970	3.490	7.580
*Prediction by RSM*			5.089	0.549	49.981	0.186	12.118	2.858	6.625
*Prediction by ANN*			5.141	0.558	49.011	0.202	16.171	3.584	7.644
Δ*_(RSM–EXP)_*			−0.026	**−0.002**	**−0.029**	**0.000**	**−0.853**	−0.632	−0.955
Δ*_(ANN–EXP)_*			0.026	0.007	−0.999	0.017	3.201	**0.094**	**0.064**
**50**	**5**	**80**							
*Average experimental data (EXP)*			5.050	0.550	40.551	0.148	9.440	2.300	7.510
*Prediction by RSM*			5.076	0.552	40.580	0.148	10.293	2.933	8.465
*Prediction by ANN*			5.230	0.545	46.683	0.171	10.730	2.262	6.042
Δ*_(RSM–EXP)_*			**0.026**	**0.002**	**0.029**	**0.000**	**0.852**	0.633	**0.955**
Δ*_(ANN–EXP)_*			0.180	−0.005	6.132	0.022	1.290	**−0.038**	−1.468
**50**	**10**	**60**							
*Average experimental data (EXP)*			5.045	0.555	83.699	0.197	10.350	1.510	14.160
*Prediction by RSM*			5.047	0.555	79.619	0.206	12.606	0.954	12.500
*Prediction by ANN*			5.097	0.545	81.463	0.199	9.765	1.486	14.157
Δ*_(RSM–EXP)_*			**0.002**	**0.001**	−4.080	0.009	2.256	−0.556	−1.660
Δ*_(ANN–EXP)_*			0.052	−0.010	**−2.236**	**0.002**	**−0.585**	**−0.024**	**−0.003**
**40**	**10**	**80**							
*Average experimental data (EXP)*			5.057	0.549	40.864	0.224	9.700	2.303	9.257
*Prediction by RSM*			5.057	0.549	40.864	0.224	9.700	2.303	9.257
*Prediction by ANN*			5.076	0.549	40.997	0.242	12.208	2.618	9.418
Δ*_(RSM–EXP)_*			**0.000**	**0.000**	**0.000**	**0.000**	**0.000**	**0.000**	**0.000**
Δ*_(ANN–EXP)_*			0.019	0.000	0.133	0.018	2.508	0.314	0.162

## Data Availability

The original contributions presented in this study are included in the article/Supplementary Materials. Further inquiries can be directed to the corresponding author.

## Supplementary Materials

The following supporting information can be downloaded at: https://www.mdpi.com/article/10.3390/foods15091507/s1, Figure S1: Calibration curve prepared from gallic acid solutions.

## Abbreviations

The following abbreviations are used in this manuscript:

ANN	Artificial Neural Network
RSM	Response Surface Methodology
TPC	Total Phenolic Content
PCI	Photometric Color Index
GAE	Gallic Acid Equivalent
MOO	Multi-objective Optimization
DOE	Design of Experiment
AOCS	American Oil Chemists’ Society
LoF	Lack-of-Fit
ANOVA	Analysis of Variance
*p*–HPEA–EA	*p*–hydroxyphenyl–ethanol–elenolic acid
DHPEA–EA	dihydroxyphenylethanol–elenolic acid
*p*–HPEA–EDA	*p*–hydroxyphenyl–ethanol–elenolic acid dialdehyde
DHPEA–EDA	dihydroxyphenylethanol–elenolic acid dialdehyde
