# Supplementary figures and images for: The Influence of Different Ultrasonication Parameters on Physicochemical Properties and Secoiridoid Compositions of Olive Extracts: A Mathematical Approach Using Artificial Neural Network (ANN) and Response Surface Methodology (RSM)
Source: Foods. 2026 Apr 26;15(9):1507. doi: 10.3390/foods15091507 (PMC13163531; doi:10.3390/foods15091507)

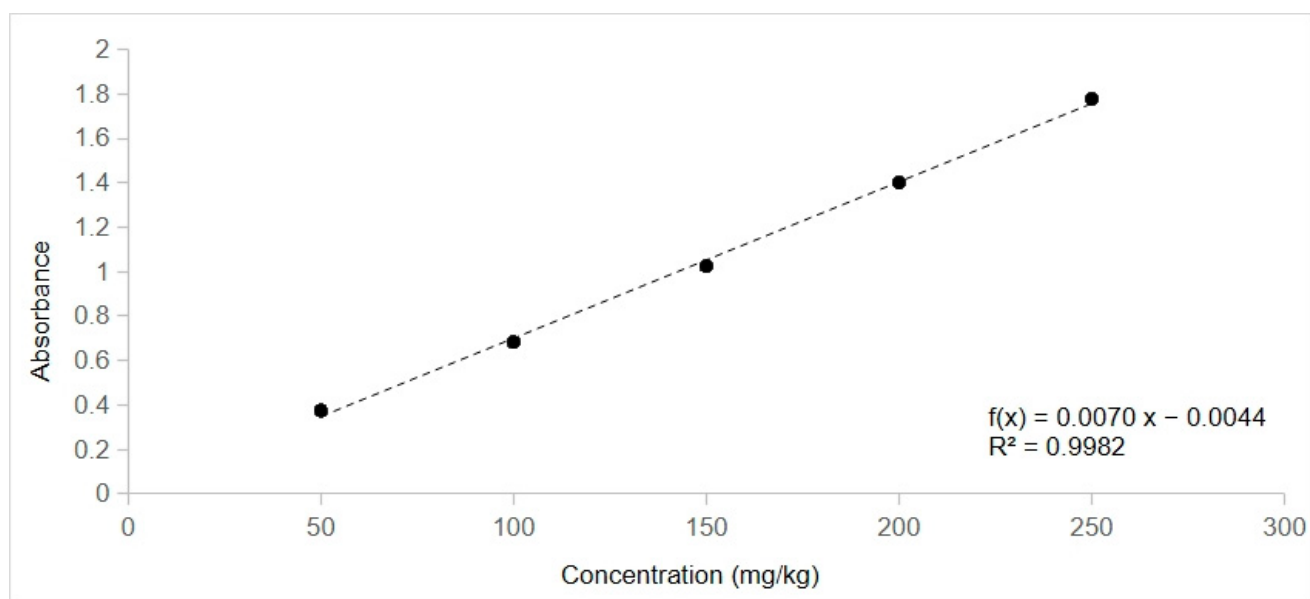

**Figure S1.** Calibration curve prepared from gallic acid solutions

Supplement: Supplementary file 1 [file foods-15-01507-s001.zip › foods-4272018-supplementary.pdf]
